# Exploring disparities: a regional analysis of harm reduction supply distribution and opioid-related deaths across Ontario’s Public Health Units

**DOI:** 10.1186/s12954-025-01319-4

**Published:** 2025-11-11

**Authors:** Shaleesa Ledlie, Kristy Scarfone, Dana Shearer, Nadia Zurba, Miroslav Miskovic, Charlotte Munro, Jes Besharah, Shauna Pinkerton, Tyler Watts, Tara Gomes

**Affiliations:** 1 Leslie Dan Faculty of Pharmacy, University of Toronto, Toronto, ON Canada; 2 Li Ka Shing Knowledge Institute, Unity Health, 30 Bond Street, Toronto, ON Canada; 3 ICES, Toronto, ON Canada; 4 Ontario Harm Reduction Distribution Program, Kingston Community Health Centres, Kingston, ON Canada; 5 Ontario Drug Policy Research Network Lived Experience Advisory Group, Toronto, ON Canada; 6 MAP Centre for Urban Health Solutions, St. Michael’s Hospital, Toronto, ON Canada; 7 Institute for Health Policy, Management, and Evaluation, University of Toronto, Toronto, ON Canada

**Keywords:** Harm reduction, Drug overdose, Naloxone, Needle exchange programs, Public health, Opioids

## Abstract

**Background:**

It is critical that a range of harm reduction supplies are available through Ontario’s Public Health Units (PHU) to meet the varying needs of people who use drugs. Therefore, we assessed geographic variation in opioid-related deaths and the distribution of these harm reduction supplies among 34 PHUs in Ontario, Canada.

**Methods:**

We conducted a population-based repeated cross-sectional study using publicly available administrative datasets between January 1, 2019, and December 31, 2022. Rates of opioid-related deaths and the distribution of harm reduction supplies (inhalation supplies, naloxone, and needles provided) were calculated per PHU. Small area rate variation statistics including the extremal quotient (EQ) were used to assess variation across PHUs in 2022.

**Results:**

Over the study period, the quarterly number of opioid-related deaths increased by 40.6% (3.2 to 4.5 per 100,000) in Ontario. The distribution rate of all harm reduction supplies increased, although there was considerable variation by type of supply. For example, the EQ ranged from 34.7 for naloxone to 1,610.6 for foil. In 2022, there were three PHUs with significantly higher rates of opioid-related deaths compared to the provincial average. In general, these PHUs also had significantly higher distribution rates of naloxone, needles, and inhalation supplies.

**Conclusions:**

Across Ontario, there is high variability in harm reduction supply distribution and opioid-related mortality. Regions with elevated opioid-related death rates also had high supply distribution rates, suggesting that efforts are concentrated in regions with particular need. To minimize harms related to substance use, ongoing efforts are needed to ensure a clear understanding of community-based needs for harm reduction services.

**Supplementary Information:**

The online version contains supplementary material available at 10.1186/s12954-025-01319-4.

## Background

Harm reduction, as it relates to substance use, first originated in North America during the 1980s when the increasing prevalence of HIV and AIDS was observed among people who inject drugs [1]. The shift from abstinence-based to person-centered interventions [2] to help mitigate harms associated with substance use led to the implementation of free needle exchange programs, with Canada’s first program launching in 1989 by Toronto Public Health [3]. These programs were highly successful in reducing the transmission of HIV and hepatitis C whilst also being cost effective [4, 5]. Furthermore, the distribution of harm reduction supplies to the community provided new opportunities for people who use drugs to discuss other health-related needs and for connections to services and supports to be established [6].

Over the past decade, Canada has continued to experience a worsening drug toxicity crisis, due to changes in the unregulated drug supply, which has increasingly shifted to being composed of fentanyl and its analogues. Over 80% of opioid-related deaths from 2020 to mid-2023 involved fentanyl, with an estimated 27,795 accidental opioid-related deaths occurring over the same period [7]. In Ontario alone, there were an estimated 2,534 lives lost to opioid-related deaths in 2022, increasing 62.5% from 2019 [7]. Correspondingly, the Ontario Ministry of Health supports the public dissemination of harm reduction supplies provincially, including naloxone kits through partnerships with pharmacies, hospitals, and community organizations in collaboration with the province’s 34 Public Health Units (PHU) [8]. Each PHU operates as an autonomous health agency, governed by local boards under legislation overseen by the Ontario Ministry of Health [9], and serves diverse populations of varying sizes (Additional file 1). Each PHU is responsible for implementing public health programs, including harm reduction, within specific geographic boundaries. These boundaries generally correspond to municipal or regional jurisdictions; however, some PHUs serve multiple municipalities, while certain larger municipalities have their own dedicated PHUs. The Ontario Naloxone Program and Ontario Naloxone Program for Pharmacies offer free naloxone kits and training to people at risk of experiencing an opioid toxicity and those who may witness a toxicity event [10]. The Ontario Harm Reduction Distribution Program (OHRDP), is a not-for-profit funded by the HIV and Hepatitis C Programs of the Ontario Ministry of Health, which coordinates the procurement, management and distribution of harm reduction supplies including sterile needles, safer smoking kits and other materials to help reduce harms related to substance use [11]. The OHRDP provides these supplies to harm reduction programs across the province including 37 core Needle Syringe Programs operating across Ontario’s PHU regions. Through these programs, all PHUs ensure access to harm reduction supplies in their local regions including access to sterile safer injection and inhalation supplies reflective of community needs [12]. These supplies are provided free of charge and without eligibility criteria, in accordance with provincial public health standards [12].

Given that varying modes of substance use (i.e., injection or inhalation) are implicated in opioid-related deaths across Ontario, it is critical that a range of harm reduction supplies are available to meet the varying needs of people who use drugs [13]. Provision of supplies and related harm reduction efforts, however, must be accessible to individuals at risk in order to be effective. This is particularly vital given that the extent of harms and primary modes of substance use, vary considerably across geographic regions in Ontario [14]. While the province’s highest numbers of opioid-related deaths generally are observed in urban centers, the highest population adjusted rates are observed within smaller rural regions where the widespread distribution of harm reduction supplies can be more challenging [15], highlighting the importance of assessing the equity in harm reduction supply distribution across all regions.

Understanding how rates of opioid-related deaths and the distribution of harm reduction supplies vary geographically across Ontario can offer important insights into the alignment between public health responses to the drug toxicity crisis and risk of harm within diverse communities. Therefore, we assessed the regional variability in the distribution of harm reduction supplies and rates of opioid-related death across Ontario, Canada.

## Methods

### Setting

We conducted a population-based repeated cross-sectional study of all accidental opioid-related deaths and harm reduction supplies distributed overall and across the 34 PHUs in Ontario, Canada, between January 1st, 2019, to December 31st, 2022. This study is reported as per the Strengthening the Reporting of Observational Studies in Epidemiology (STROBE) guidelines.

### Data sources

We obtained publicly available aggregate data from the Ontario Drug Policy Research Network (ODPRN)’s Opioid Indicator Tool [16], and Public Health Ontario (PHO)’s Interactive Opioid Tool [17]. These tools contain aggregate summaries of data from several different sources, including the Office of the Chief Coroner of Ontario, the Ontario Drug Benefit Database, the Ontario Ministry of Health, and the Registered Persons Database. Specifically, we identified quarterly counts of opioid-related deaths from PHO’s Interactive Opioid Tool. In Ontario, a medical coroner investigates all sudden or unexpected deaths (including suspected drug toxicity deaths), with PHO’s tool capturing all such deaths in Ontario where it was determined that an opioid directly contributed to death and that the death was unintentional. We identified quarterly counts of pharmacy and community-dispensed naloxone, straight stems, bowl pipes, straws, foil, and needles provided through the Ontario Naloxone Program, the Ontario Naloxone Program for Pharmacies, Ontario Harm Reduction Distribution Program, and Needle Exchange Program using data from the ODPRN’s Opioid Indicator Tool. Naloxone kits are dispensed through participating pharmacies and community-based organizations affiliated with PHUs. The pharmacy data captures naloxone dispensed directly to individuals, while community distribution data reflects supplies provided through PHU-led harm reduction services. We also identified the population of Ontario and people residing within each PHU on a quarterly basis from the ODPRN’s Opioid Indicator Tool, which uses the Registered Persons Database in Ontario, to reflect all individuals residing in the province during these periods who have issued a health card.

### Study measures

Our study measures included rates of opioid-related deaths and the distribution of harm reduction supplies in Ontario. These harm reduction supplies included naloxone doses, needles, straight stems, foil, straws, and bowl pipes. Each item serves a specific purpose in helping to reduce the harms associated with drug use. Needles are intended for injecting drugs, straight stems are intended for smoking or vaping drugs, foil is used to smoke or vape substances that release inhalable vapours when heated, straws are used either for snorting powdered drugs or inhaling vapour produced by heating drugs on foil, and bowl pipes are designed for smoking drugs in crystal form by placing the crystals at the bottom of the bowl and heating them to produce vapor [18]. Naloxone is used to reverse opioid toxicities and is available in both intranasal and injectable forms through both pharmacies and community-based initiatives. We used the population of Ontario and within each PHU to calculate population-adjusted rates for each type of harm reduction supply per 1,000 population and opioid-related deaths per 100,000 population and reported all measures overall and stratified by PHU.

### Statistical analysis

In our primary analysis, we examined provincial trends in rates of harm reduction supply distribution and opioid-related deaths quarterly across the study period. The distribution of straws did not begin until the first quarter of 2020 and therefore data on this indicator is limited to this period onwards. Needle Exchange Program data was not available on a quarterly basis and was therefore excluded from our primary analysis.

We also conducted three secondary analyses to assess variation in our study measures among PHUs, restricted to the final year of the study period (2022). First we conducted a small area rate variation (SARV) analysis to identify variation in the distribution rates of harm reduction supplies across all PHUs [19, 20]. We used the extremal quotient (EQ) to describe the observed ratio between the highest and lowest PHU dispensing rate for each harm reduction supply and the coefficient of variation (CV) to describe the relative variability among each type of harm reduction supply. Next, we conducted individual chi-square tests to compare the rates of harm reduction supply distribution and opioid-related deaths among each PHU, compared to the provincial average. We applied the Bonferroni correction to limit the likelihood of false positive error rate due to multiple comparisons and therefore used a type I error rate of *p* ≤ 0.001 (*p* = 0.05/34). Finally, we calculated the location quotient (LQ) with corresponding 95% confidence intervals (CI) for each PHU, to describe the regional concentration of harm reduction supply distribution compared to the provincial average. Location quotient values greater than one indicate PHUs with rates higher than the provincial average, while values less than one indicate rates lower than the provincial average [21]. There were four PHUs that did not order straws in 2022, and therefore data on straws were only included in our chi-square analysis. All analyses were completed using Excel (Version 16.78.3), RStudio (Version 2022.12.0 + 353) and SAS version 9.4 (SAS institute, Cary, North Carolina, USA).

### Involvement of people with lived and living experience

The ODPRN’s research is informed by a Lived Experience Advisory Group, comprised of individuals with lived and living experience using opioids who were involved throughout the design of the ODPRN’s Opioid Indicator Tool (the data used in this study). Members of the Lived Experience Advisory Group also participated as study team members on this study, providing feedback on the contextualization and reporting of results. All people with lived and living experience were compensated for their time spent on this study and were offered co-authorship (or when preferred by the individual, acknowledgment) in this manuscript.

### Ethics approval

Research ethics approval was not required for this study as all data are anonymized and publicly available in aggregate form on the forementioned online tools.

## Results

**Fig. 1:**
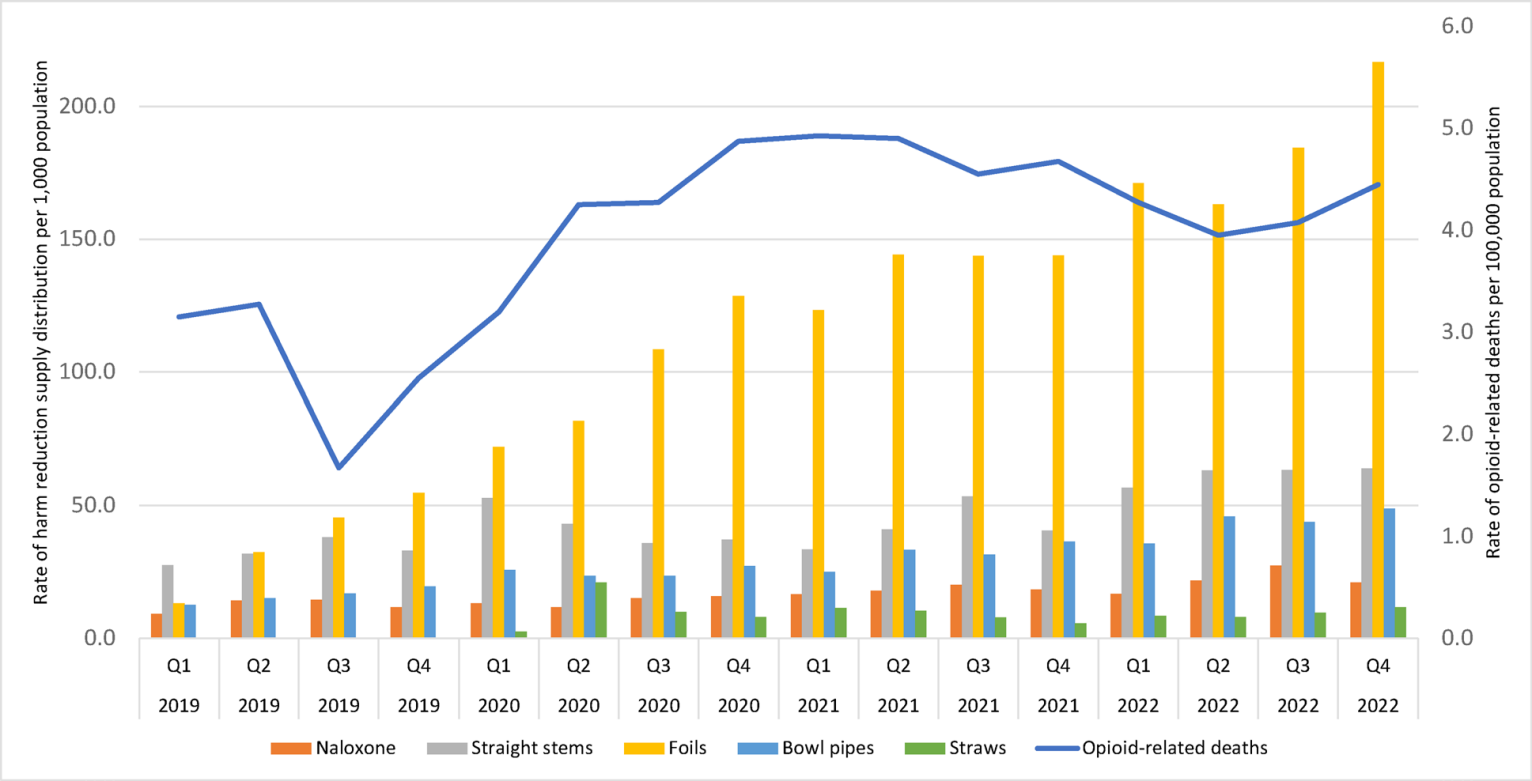
Quarterly rates of harm reduction supply distribution and opioid-related deaths across Ontario. Note: Data on the distribution of straws was limited from Q1 2020 onwards

Over the study period, we observed 9,412 opioid-related deaths, with the quarterly number of deaths increasing by 40.6% from 458 (3.2 per 100,000) in the first quarter of 2019 to 678 (4.5 per 100,000) in the fourth quarter of 2022. Over the same period, 3,913,838 naloxone doses, 10,537,560 straight stems, 6,860,592 bowl pipes, 1,689,500 straws, and 26,982,000 foil were provided provincially. In general, the distribution of harm reduction supplies increased over the study period, although the magnitude of this increase varied by type of harm reduction supply. For instance, we observed a 126.9% increase in the rate of naloxone doses (9.3–21.1 per 1,000), a 132.7% increase in straight stems (27.5–64.0 per 1,000), a 287.3% increase in bowl pipes (12.6–48.8 per 1,000), a 346.2% increase in straws (2.6–11.6 per 1,000), and a 1,540.9% increase in foil (13.2–216.6 per 1,000) distributed provincially, quarterly, over the study period. Quarterly trends in the distribution rates of harm reduction supplies and opioid-related deaths can be found in Fig. 1 and Additional file 2.

**Fig. 2:**
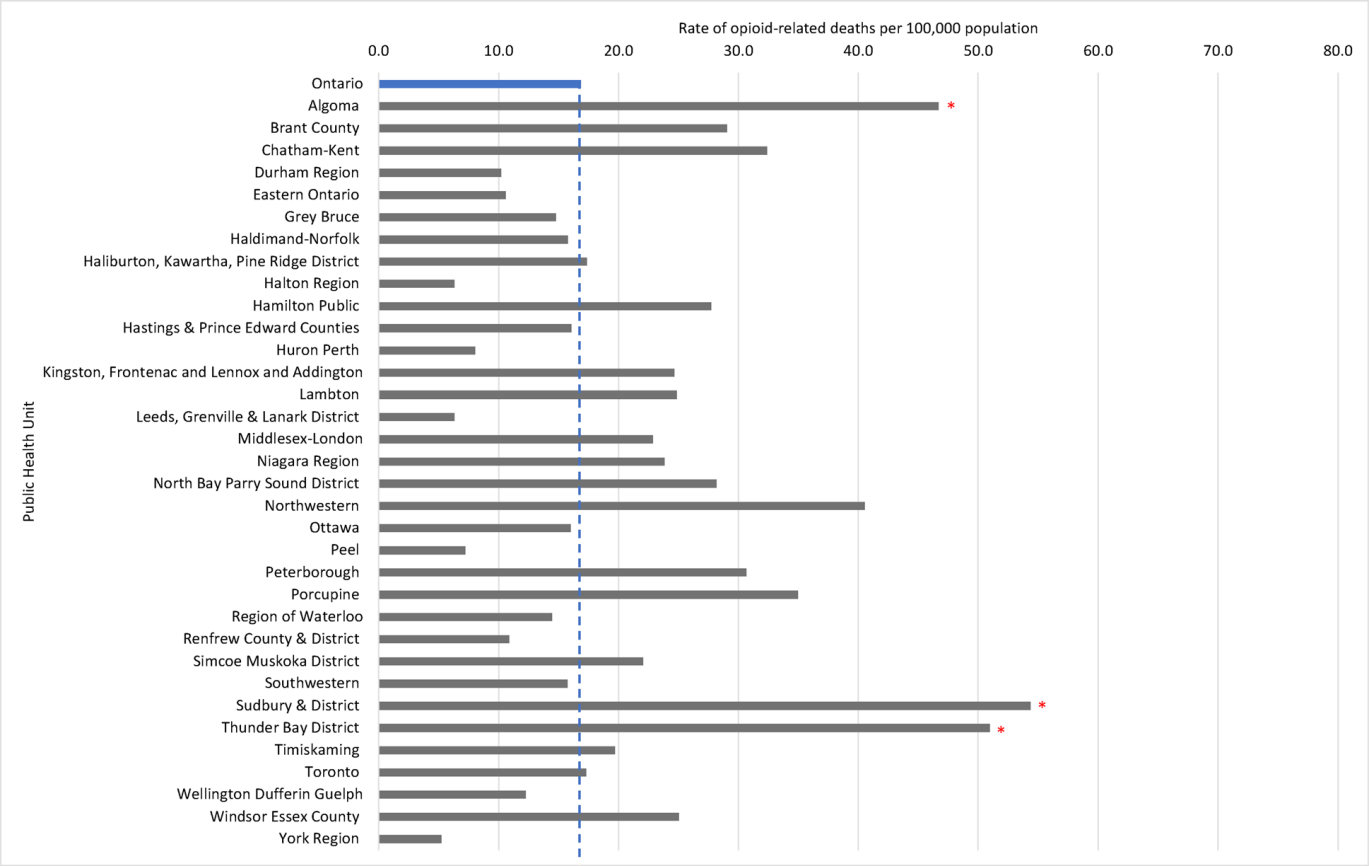
Opioid-related death rates across Ontario and within each Public Health Unit, 2022. Note: The Ontario average rate of opioid-related deaths is marked by the vertical blue line. * Denotes PHUs with significantly higher (p≤.001) opioid-related death rates compared to the average across Ontario

We observed significant variation in the rate of opioid-related deaths and the rate of distribution of harm reduction supplies among PHUs in 2022. Results from our SARV analysis highlighted that the magnitude of this variation differed among types of harm reduction supplies (Table 1). For instance, the EQ (which measures the ratio of highest and lowest rates) ranged from 34.7 for naloxone to 1,610.6 for foil, indicating that considerable variation exists among PHUs in the distribution of harm reduction supplies intended for inhalation use, as compared to a relatively low geographic variation in the distribution of naloxone. Similar results were observed with the CV (which measures the degree of dispersion of each rate from the provincial average) which ranged from 0.8 for naloxone to 1.4 for needles provided, indicating that the geographic distribution of naloxone is more consistent compared to needles. In our regional analysis, we identified three PHUs (Algoma Public Health, Sudbury & District Public Health, and Thunder Bay District) with significantly higher rates of opioid-related deaths compared to the provincial average (Fig. 2). With few exceptions, these three PHUs also had significantly higher distribution rates of all harm reduction supplies compared to the provincial average (Table 2). Additionally, other PHUs with moderate opioid-related death rates had elevated distribution rates of specific types of harm reduction supplies such as Northwestern Health where we observed the highest rate of needles provided in 2022 (16,292.9 per 1,000 population). In contrast, PHUs with low rates of opioid-related deaths (e.g., York Region and Halton Region) generally also had lower distribution rates of harm reduction supplies. Finally, we observed some PHUs (e.g., Durham Region and Peel Region) with comparable rates of opioid-related deaths to the provincial average where there were significantly lower distribution rates of various harm reduction supplies. Our findings were generally consistent between our chi-squared tests and calculation of the LQ among each PHU (Additional file 3). A map of opioid-related death rates across PHUs can be found in Additional file 4.

**Table 1:** Small area rate variation analysis of distributed harm reduction supplies across all Public Health Units, 2022

Harm reduction supply	Extremal quotient	Coefficient of variation
Needles	229.9	1.4
Straight stems	173.3	1.0
Foil	1,610.6	1.1
Naloxone	34.7	0.8
Bowl pipes	76.0	0.9
Straws	253.5	1.1

**Table 2:**
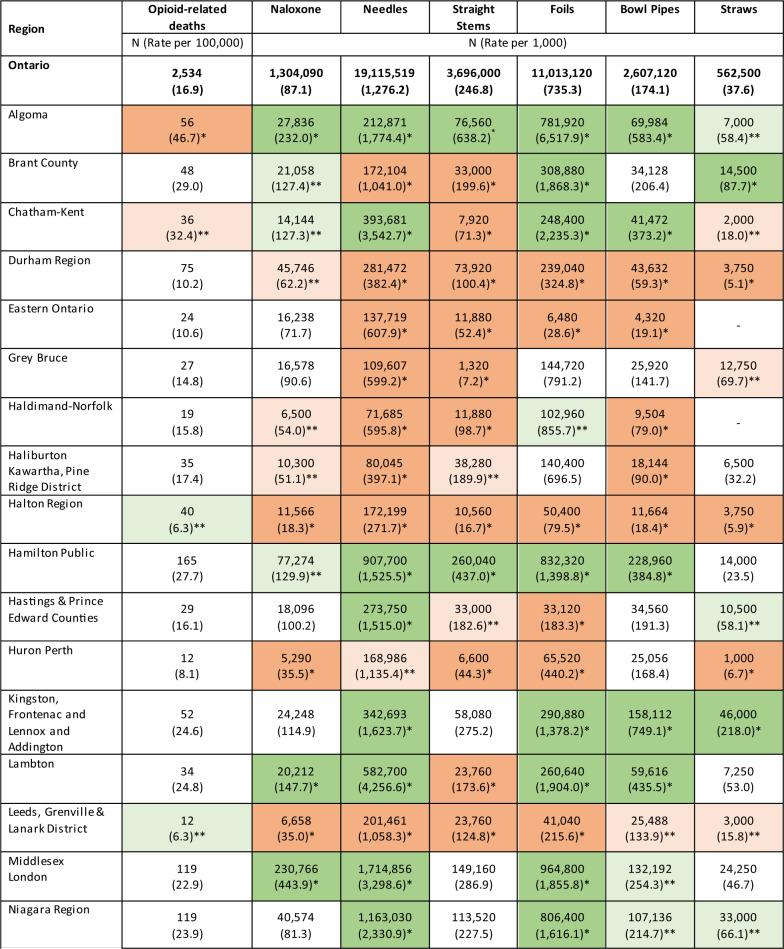

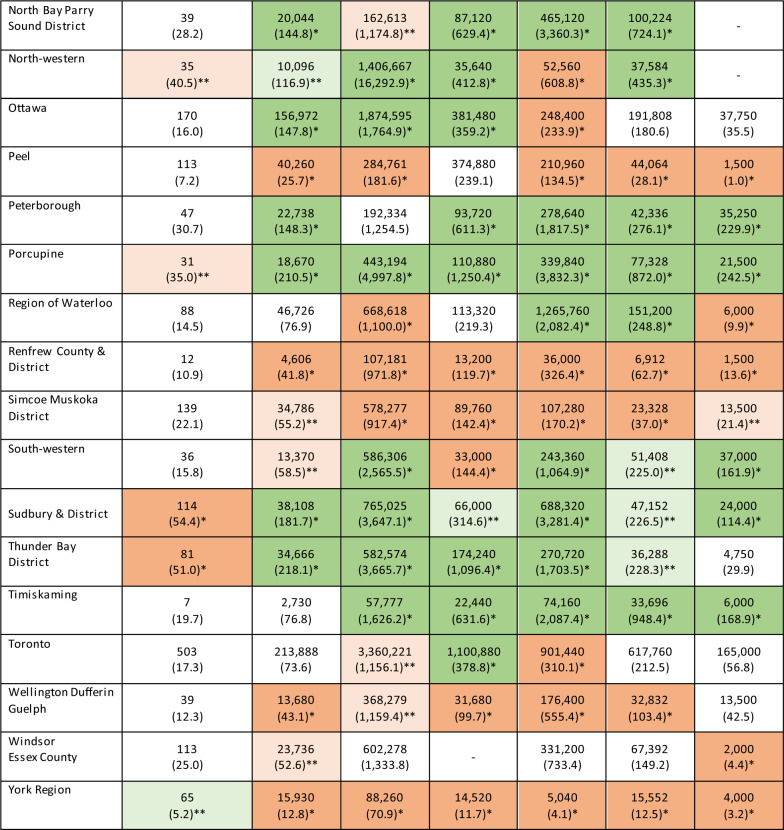
Results from the chi-square test comparing rates of opioid-related deaths and harm reduction supply distribution among each Public Health Unit and Ontario, 2022

*Denotes PHUs with rates which significantly differ (Bonferroni corrected, *p*≤.001) from the Ontario average—dark green (higher rate compared to the provincial average) and dark red (lower rate compared to the provincial average)

**Denotes PHUs with rates which significantly differ (non-Bonferroni corrected, *p*<.05) from the Ontario average–light green (higher rate compared to the provincial average) and light red (lower rate compared to the provincial average)

## Discussion

Over a three-year period, the distribution rates of harm reduction supplies increased dramatically across Ontario, in parallel with growing rates of opioid-related deaths. We observed substantial variation across PHUs in rates of opioid-related death and in the distribution of harm reduction supplies. In some PHUs, we found that high rates of opioid-related death were accompanied by high distribution rates of harm reduction supplies, while in other regions we observed low rates of opioid-related deaths coupled with low harm reduction distribution rates. Although harm reduction supplies, with the exception of naloxone, do not necessarily reduce opioid-related death, they do help to prevent other harms associated with substance use (e.g., injection-related infections). Therefore, our findings likely reflect active region-specific responses to the ongoing needs of people who use drugs and highlights that, in general, there may be fewer available harm reduction services in communities with historically lower rates of harms related to substance use and where people may be able to access harm reduction services in neighboring regions. Furthermore, high distribution rates of harm reduction supplies intended for either inhalation or injection in some PHUs, may reflect local preferences for specific modes of drug use.

Our findings add to the growing body of literature characterizing trends in mode of drug use among people who use drugs [22, 23], as we anticipate that PHU-level differences in the distribution of harm reduction supplies may be related to preferred modes of drug use within each region. For instance, evidence from Ontario’s coronial data has suggested that the mode of drug use shifted considerably toward inhalation between 2019 and 2021, with a 64.4% decrease observed in deaths with indication of injection alone [22]. Interviews conducted with people who use drugs found that motivations for inhalation as a preferred mode of substance use include a lower perceived risk of overdose and the desire to avoid potential injection-related complications [24, 25]. This preference may also align with the increasing use of stimulants, particularly in combination with opioids. Between 2018 and 2021, rates of stimulant toxicity deaths in Ontario rose from 0.4 to 1.0 per 100,000, with over 80% of these deaths also involving an opioid [26]. The rise in the distribution of harm reduction supplies associated with inhalation may therefore reflect not only shifting modes of use but also an increase in the use of stimulants where inhalation is commonly the preferred method of administration [27]. This also aligns with data from the British Columbia Coroner’s Service in 2021, where a 20% decrease was observed in drug toxicity deaths involving injection drug use, alongside a 56% increase in deaths involving inhalation [23]. Therefore, the growing rates of distribution of bowl pipes and foil (which increased 287.3% and 1,540.9%, respectively) in this study likely parallel changing patterns of substance use across the province, leading to an increased demand for harm reduction supplies used for smoking and inhalation. Furthermore, we found that in general, PHUs with the highest distribution rates of inhalation supplies were those with a high proportion of opioid-related deaths involving inhalation [14]. For example, Algoma Public Health, a region with the highest proportion of opioid-related deaths involving inhalation across Ontario [14], had significantly higher rates of distribution of all types of inhalation supplies. In comparison, although there was some alignment in needles provided and opioid-related deaths involving injection drug use, gaps existed in some regions where reports [14] have shown relatively high indicators of injection drug use but only moderate levels of distribution of needles through Needle Exchange Programs. This highlights the importance of community engagement with people who use drugs to identify local preferred modes of drug use to help identify the types of harm reduction supplies and programs that should be concentrated in different geographic regions.

The finding that three PHUs with rates of opioid-related deaths significantly greater than the provincial average are in Northern Ontario is also important, as these geographic areas often have large rural and remote regions with dispersed populations that can make the distribution of harm reduction supplies and provision of treatment services more complex to deliver [28, 29]. Interviews conducted with youth who use drugs residing in Northern Ontario identified several structural barriers to accessing substance-related treatment including long wait lists, extremely under-resourced services with limited capacity to take new clients, a lack of personal transportation coupled with poor public transit, and the requirement to travel to neighboring communities to access services [30]. Regions of Northern Ontario are home to more Indigenous, majority being First Nation communities, highlighting the importance of furthering efforts for equitable, accessible, Indigenous-led, culturally responsive harm reduction programs and services that meet the needs of Indigenous Peoples inclusive of populations residing in rural and remote communities [31]. An example is the recent implementation of mobile crisis response and harm reduction outreach teams in some Northern Ontario communities that are able to assist individuals with complex needs who may otherwise have difficulties accessing the healthcare system [32, 33]. Continued funding is required to scale-up harm reduction programs that are tailored to and focused on equitable access in these regions, and in other remote parts of the province.

Given the nature of the unregulated drug supply, the distribution of harm reduction supplies alone is insufficient to address the current drug toxicity crisis. It is therefore critical that access to these supplies is coupled with the expansion of other evidence-based harm reduction programs including drug-checking services, treatment for substance use disorders, safer opioid supply programs and supervised consumption sites that can also help to reduce the harms associated with substance use. For example, a recent study conducted in Toronto, Ontario found that within neighbourhoods that implemented a supervised consumption site, there was a 67% reduction in rates of opioid-related deaths [34]. Supervised consumption sites also play an important role in the distribution of harm reduction supplies by providing people who use drugs with a safe, non-judgmental environment and sterile consumption equipment [35]. Given that a large majority of these sites are currently located within large, urban regions such as Toronto and Ottawa [36], the continued funding of supervised consumption sites across Ontario is warranted, particularly in regions where access to harm reduction services is limited.

This study has several limitations that warrant discussion. First, due to the nature of the available data, covariates including age and sex were not available to standardize population rates. Likewise, no data was available related to person-level risk factors for substance-related harms because harm reduction supplies are provided to regional programs for broader distribution and are therefore not tracked at an individual level. Further research is required to investigate the relationship between distribution rates of harm reduction supplies and various person, program and geographic-related factors within each PHU including the number of staff and availability of adequate resources to distribute harm reduction supplies. Second, harm reduction supply distribution rates reflect the provision of these products from the Ministry of Health to each PHU for distribution in the community, and as such, the timing at which supplies are distributed to the community may vary. Further, the counts of naloxone doses provided do not account for what is released to emergency services such as police, fire, or St. John’s Ambulance as those doses are intended for administration in emergency scenarios and not intended to be provided directly to the public for individual use. Similarly, the counts of needles and syringes reported in Northwestern Health are under-reported due to the independent purchasing of these supplies in some parts of the region. However, we anticipate that the exclusion of these counts has limited impact on the interpretation of our results given our focus on community-based distribution of harm reduction supplies. Third, this study was not designed to determine the impact of harm reduction supply distribution on rates of opioid-related deaths, including whether high regional rates of harm reduction supply distribution were correlated with low opioid-related death rates as the distribution of harm reduction supplies (apart from naloxone) reduces the harms associated with substance use but does not directly prevent overdose deaths. We were also unable to assess if PHUs with low opioid-related death rates and low rates of harm reduction supply distribution were still providing accessible harm reduction to those in need. Finally, we relied on data from the Chief Coroner of Ontario to estimate patterns in modes of substance use and as such did not have access to complete data on mode of substance use preferences within each PHU, which may influence the distribution rate of different harm reduction supplies. Given that these harm reduction supplies are also used for non-opioid substances (including tobacco), future work is needed to better understand mode of use patterns for other substances.

## Conclusions

Across Ontario, there is significant variability in opioid-related deaths, underscoring the need for regional-level responses that incorporate the distribution of harm reduction supplies with improved access to treatment and other targeted harm-reduction interventions. We found considerable variability in the distribution of harm-reduction supplies, which suggest that public health efforts are being concentrated in PHUs with higher rates of opioid-related deaths and may be tailored to common modes of drug use within communities. Given the widespread nature of harms, ongoing efforts are needed to clearly understand specific community-based needs for harm reduction services and supplies to help ensure that provision of supplies and approaches to distribution are responsive to community needs and adapted to unique needs of more rural and remote parts of the province.

## Supplementary Information

Additional file.

## Data Availability

The data used in this study are publicly available from the Ontario Drug Policy Research Network and Public Health Ontario’s respective online tools. Program files can be made available by reasonable request to the corresponding author.

## Abbreviations

CV: Coefficient of variation
CI: Confidence interval
EQ: Extremal quotient
LQ: Location quotient
ODPRN: Ontario Drug Policy Research Network
OHRDP: Ontario Harm Reduction Distribution Program
PHO: Public Health Ontario
PHU: Public Health Units
SARV: Small area rate variation
STROBE: Strengthening the Reporting of Observational Studies in Epidemiology
