# Supplementary material for: Exploring disparities: a regional analysis of harm reduction supply distribution and opioid-related deaths across Ontario’s Public Health Units
Source: Harm Reduct J. 2025 Nov 11;22:184. doi: 10.1186/s12954-025-01319-4 (PMC12606891; doi:10.1186/s12954-025-01319-4)

**ADDITIONAL FILES**

**Additional file 1.** Characteristics of Ontario Public Health Units (PHUs), Including Population Estimates and Total Private Dwellings from the 2021 Statistics Canada Census

| **Public Health Unit Name** | **Population (2021)** | **Total Private Dwellings (2021)** |
| --- | --- | --- |
| Algoma | 112,764 | 59,245 |
| Brant County | 144,937 | 58,308 |
| Chatham-Kent | 104,316 | 46,921 |
| Durham Region | 696,992 | 250,559 |
| Eastern Ontario | 210,276 | 90,418 |
| Grey Bruce | 174,301 | 92,775 |
| Haldimand-Norfolk | 116,706 | 50,795 |
| Haliburton Kawartha, Pine Ridge District | 189,183 | 100,657 |
| Halton Region | 596,637 | 214,322 |
| Hamilton Public Health | 569,353 | 233,564 |
| Hastings & Prince Edward Counties | 171,450 | 82,075 |
| Huron Perth | 142,931 | 63,517 |
| Kingston, Frontenac and Lennox and Addington | 206,962 | 100,320 |
| Lambton | 128,154 | 60,322 |
| Leeds, Grenville & Lanark District | 179,830 | 84,998 |
| Middlesex London | 500,563 | 216,736 |
| Niagara Region | 477,941 | 207,926 |
| North Bay Parry Sound District | 129,362 | 75,352 |
| North-western | 77,338 | 41,342 |
| Ottawa | 1,017,449 | 427,113 |
| Peel | 1,451,022 | 467,970 |
| Peterborough | 147,681 | 73,045 |
| Porcupine | 81,188 | 39,038 |
| Region of Waterloo | 587,165 | 233,253 |
| Renfrew County & District | 107,522 | 52,365 |
| Simcoe Muskoka District | 599,843 | 272,647 |
| South-western | 216,533 | 88,334 |
| Sudbury & District | 202,431 | 98,791 |
| Thunder Bay District | 152,885 | 74,175 |
| Timiskaming | 32,394 | 17,145 |
| Toronto | 2,794,356 | 1,253,238 |
| Wellington Dufferin Guelph | 307,283 | 121,675 |
| Windsor Essex County | 422,860 | 174,446 |
| York Region | 1,173,334 | 405,863 |

**Additional file 2.** Rates of opioid-related deaths and harm reduction supply distribution by quarter across Ontario.

| **Year** | **Quarter** | **Opioid-related deaths** | | **Naloxone** | | **Straight stems** | | **Bowl pipes** | | **Straws** | | **Foil** | |
| --- | --- | --- | --- | --- | --- | --- | --- | --- | --- | --- | --- | --- | --- |
|  |  | N | Rate^*^ | N | Rate^†^ | N | Rate^†^ | N | Rate^†^ | N | Rate^†^ | N | Rate^†^ |
| 2019 | Q1 | 458 | 3.2 | 133,856 | 9.3 | 394,680 | 27.5 | 181,008 | 12.6 | N/A | N/A | 190,080 | 13.2 |
|  | Q2 | 479 | 3.3 | 204,126 | 14.2 | 459,360 | 31.9 | 216,864 | 15.0 | N/A | N/A | 467,280 | 32.4 |
|  | Q3 | 247 | 1.7 | 210,160 | 14.5 | 550,440 | 38.0 | 244,080 | 16.9 | N/A | N/A | 658,080 | 45.5 |
|  | Q4 | 375 | 2.6 | 169,232 | 11.6 | 481,800 | 33.1 | 285,120 | 19.6 | N/A | N/A | 795,600 | 54.7 |
| 2020 | Q1 | 471 | 3.2 | 191,092 | 13.1 | 769,560 | 52.8 | 374,976 | 25.7 | 37,500 | 2.6 | 1,049,040 | 72.0 |
|  | Q2 | 630 | 4.3 | 169,896 | 11.6 | 627,000 | 43 | 341,712 | 23.4 | 306,000 | 21 | 1,193,040 | 81.8 |
|  | Q3 | 635 | 4.3 | 220,348 | 15.0 | 525,360 | 35.9 | 344,736 | 23.5 | 145,250 | 9.9 | 1,589,040 | 108.5 |
|  | Q4 | 725 | 4.9 | 231,586 | 15.8 | 546,480 | 37.2 | 400,032 | 27.2 | 117,500 | 8.0 | 1,888,560 | 128.6 |
| 2021 | Q1 | 736 | 4.9 | 243,774 | 16.6 | 491,040 | 33.4 | 368,064 | 25.0 | 167,250 | 11.4 | 1,815,120 | 123.4 |
|  | Q2 | 733 | 4.9 | 265,000 | 18.0 | 604,560 | 41.0 | 491,184 | 33.3 | 154,750 | 10.5 | 2,059,920 | 144.0 |
|  | Q3 | 684 | 4.6 | 298,638 | 20.2 | 789,360 | 53.3 | 466,128 | 31.5 | 114,750 | 7.8 | 2,129,760 | 143.8 |
|  | Q4 | 704 | 4.7 | 272,040 | 18.4 | 601,920 | 40.6 | 539,568 | 36.4 | 84,000 | 5.7 | 2,133,360 | 143.9 |
| 2022 | Q1 | 644 | 4.3 | 250,832 | 16.8 | 842,160 | 56.5 | 531,360 | 35.7 | 123,500 | 8.3 | 2,547,360 | 171.0 |
|  | Q2 | 596 | 4.0 | 324,066 | 21.7 | 942,480 | 63.0 | 685,152 | 45.8 | 120,250 | 8.0 | 2,438,640 | 163.1 |
|  | Q3 | 617 | 4.1 | 412,252 | 27.5 | 949,080 | 63.2 | 656,208 | 43.7 | 144,750 | 9.6 | 2,769,120 | 184.4 |
|  | Q4 | 678 | 4.5 | 316,940 | 21.1 | 962,280 | 64.0 | 734,400 | 48.8 | 174,000 | 11.6 | 3,258,000 | 216.6 |

^*Rate expressed per 100,000; † rates expressed per 1,000.^

**Additional file 3.** Harm reduction supply distribution location quotient (LQ) in each Public Health Unit, 2022.

| **Public Health Unit** | **Naloxone** | | **Needles** | | **Straight Stems** | | **Foil** | | **Bowl Pipes** | | **Straws** | |
| --- | --- | --- | --- | --- | --- | --- | --- | --- | --- | --- | --- | --- |
|  | LQ | 95% CI | LQ | 95% CI | LQ | 95% CI | LQ | 95% CI | LQ | 95% CI | LQ | 95% CI |
| Algoma | 2.67 | 2.34, 3.02 | 1.39 | 1.33, 1.46 | 2.59 | 2.39, 2.79 | 8.86 | 8.65, 9.08 | 3.35 | 3.09, 3.63 | 1.55 | 1.19, 1.99 |
| Brant County | 1.46 | 1.22, 1.73 | 0.82 | 0.77, 0.87 | 0.81 | 0.70, 0.93 | 2.54 | 2.43, 2.66 | 1.19 | 1.03, 1.36 | 2.34 | 1.88, 2.86 |
| Chatham-Kent | 1.46 | 1.22, 1.73 | 2.78 | 2.69, 2.87 | 0.29 | 0.23, 0.36 | 3.04 | 2.92, 3.17 | 2.14 | 1.93, 2.37 | 0.48 | 0.29, 0.74 |
| Durham Region | 0.71 | 0.55, 0.91 | 0.30 | 0.27, 0.33 | 0.41 | 0.33, 0.49 | 0.44 | 0.40, 0.49 | 0.34 | 0.26, 0.43 | 0.14 | 0.05, 0.29 |
| Eastern Ontario | 0.82 | 0.65, 1.03 | 0.48 | 0.44, 0.52 | 0.21 | 0.16, 0.28 | 0.04 | 0.03, 0.05 | 0.11 | 0.067, 0.17 | - | - |
| Grey Bruce | 1.04 | 0.84, 1.27 | 0.47 | 0.43, 0.51 | 0.03 | 0.01, 0.06 | 1.08 | 1.00, 1.15 | 0.81 | 0.69, 0.96 | 1.86 | 1.45, 2.33 |
| Haldimand-Norfolk | 0.62 | 0.47, 0.80 | 0.47 | 0.43, 0.51 | 0.40 | 0.33, 0.48 | 1.16 | 1.09, 1.24 | 0.45 | 0.36, 0.56 | - | - |
| Haliburton, Kawartha, Pine Ridge District | 0.59 | 0.44, 0.76 | 0.31 | 0.28, 0.34 | 0.77 | 0.67, 0.88 | 0.95 | 0.88, 1.02 | 0.52 | 0.42, 0.63 | 0.86 | 0.60, 1.19 |
| Halton Region | 0.21 | 0.13, 0.32 | 0.21 | 0.19, 0.24 | 0.07 | 0.04, 0.11 | 0.11 | 0.09, 0.13 | 0.11 | 0.064, 0.16 | 0.16 | 0.06, 0.32 |
| Hamilton | 1.49 | 1.25, 1.76 | 1.20 | 1.14, 1.26 | 1.77 | 1.61, 1.94 | 1.90 | 1.80, 2.00 | 2.21 | 2.0, 2.44 | 0.63 | 0.41, 0.91 |
| Hastings and Prince Edward Counties | 1.15 | 0.94, 1.39 | 1.19 | 1.13, 1.25 | 0.74 | 0.64, 0.85 | 0.25 | 0.21, 0.29 | 1.10 | 0.95, 1.26 | 1.55 | 1.18, 1.98 |
| Huron Perth | 0.41 | 0.29, 0.56 | 0.89 | 0.84, 0.94 | 0.18 | 0.13, 0.24 | 0.60 | 0.54, 0.66 | 0.97 | 0.83, 1.12 | 0.18 | 0.08, 0.35 |
| Kingston, Frontenac and Lennox and Addington | 1.32 | 1.09, 1.58 | 1.27 | 1.21, 1.34 | 1.12 | 0.99, 1.25 | 1.87 | 1.8, 1.98 | 4.30 | 4.00, 4.62 | 5.80 | 5.07, 6.61 |
| Lambton | 1.70 | 1.44, 1.98 | 3.34 | 3.24, 3.44 | 0.70 | 0.60, 0.81 | 2.59 | 2.47, 2.71 | 2.50 | 2.27, 2.74 | 1.41 | 1.06, 1.83 |
| Leeds, Grenville, and Lanark District | 0.40 | 0.28, 0.55 | 0.83 | 0.78, 0.88 | 0.51 | 0.42, 0.60 | 0.29 | 0.26, 0.33 | 0.77 | 0.65, 0.91 | 0.42 | 0.25, 0.66 |
| Middlesex-London | 5.10 | 4.64, 5.59 | 2.58 | 2.50, 2.67 | 1.16 | 1.03, 1.30 | 2.52 | 2.41, 2.64 | 1.46 | 1.29, 1.65 | 1.24 | 0.92, 1.63 |
| Niagara Region | 0.93 | 0.75, 1.15 | 1.83 | 1.75, 1.90 | 0.92 | 0.81, 1.05 | 2.20 | 2.09, 2.31 | 1.23 | 1.08, 1.41 | 1.76 | 1.37, 2.22 |
| North Bay Parry Sound District | 1.66 | 1.41, 1.95 | 0.92 | 0.87, 0.97 | 2.55 | 2.36, 2.76 | 4.57 | 4.42, 4.73 | 4.16 | 3.86, 4.47 | - | - |
| Northwestern | 1.34 | 1.11, 1.60 | 12.77 | 12.57, 12.96 | 1.67 | 1.52, 1.84 | 0.83 | 0.76, 0.90 | 2.50 | 2.27, 2.74 | - | - |
| Ottawa | 1.70 | 1.44, 1.99 | 1.38 | 1.32, 1.45 | 1.46 | 1.31, 1.61 | 0.32 | 0.28, 0.36 | 1.04 | 0.89, 1.20 | 0.95 | 0.67, 1.29 |
| Peel | 0.29 | 0.20, 0.42 | 0.14 | 0.12, 0.16 | 0.97 | 0.85, 1.10 | 0.18 | 0.15, 0.22 | 0.16 | 0.11, 0.23 | 0.03 | 0.001, 0.11 |
| Peterborough | 1.70 | 1.44, 1.99 | 0.98 | 0.93, 1.04 | 2.48 | 2.29, 2.68 | 2.47 | 2.36, 2.59 | 1.59 | 1.41, 1.78 | 6.12 | 5.37, 6.95 |
| Porcupine | 2.42 | 2.11, 2.76 | 3.92 | 3.81, 4.03 | 5.07 | 4.79, 5.35 | 5.21 | 5.05, 5.38 | 5.01 | 4.68, 5.35 | 6.46 | 5.68, 7.30 |
| Region of Waterloo | 0.88 | 0.70, 1.10 | 0.86 | 0.81, 0.91 | 0.89 | 0.78, 1.01 | 2.83 | 2.71, 2.96 | 1.43 | 1.26, 1.61 | 0.26 | 0.13, 0.46 |
| Renfrew County and District | 0.48 | 0.35, 0.64 | 0.76 | 0.71, 0.81 | 0.49 | 0.40, 0.58 | 0.44 | 0.40, 0.49 | 0.36 | 0.28, 0.46 | 0.36 | 0.20, 0.59 |
| Simcoe Muskoka District | 0.63 | 0.48, 0.82 | 0.72 | 0.67, 0.77 | 0.58 | 0.49, 0.68 | 0.23 | 0.20, 0.27 | 0.21 | 0.15, 0.29 | 0.57 | 0.36, 0.85 |
| Southwestern | 0.67 | 0.51, 0.86 | 2.01 | 1.93, 2.09 | 0.59 | 0.49, 0.69 | 1.45 | 1.36, 1.54 | 1.29 | 1.13, 1.47 | 4.31 | 3.68, 5.01 |
| Sudbury and District | 2.09 | 1.80, 2.41 | 2.86 | 2.77, 2.95 | 1.28 | 1.14, 1.42 | 4.46 | 4.31, 4.62 | 1.30 | 1.14, 1.48 | 3.05 | 2.52, 3.64 |
| Thunder Bay District | 2.51 | 2.19, 2.85 | 2.87 | 2.78, 2.97 | 4.44 | 4.19, 4.71 | 2.32 | 2.21, 2.43 | 1.31 | 1.15, 1.49 | 0.80 | 0.54, 1.13 |
| Timiskaming | 0.88 | 0.70, 1.10 | 1.27 | 1.21, 1.34 | 2.56 | 2.37, 2.76 | 2.84 | 2.72, 2.96 | 5.45 | 5.11, 5.80 | 4.50 | 3.85, 5.21 |
| Toronto | 0.85 | 0.67, 1.05 | 0.91 | 0.85, 0.96 | 1.53 | 1.39, 1.69 | 0.42 | 0.38, 0.47 | 1.22 | 1.06, 1.39 | 1.51 | 1.15, 1.94 |
| Wellington-Dufferin-Guelph | 0.49 | 0.36, 0.66 | 0.91 | 0.86, 0.96 | 0.40 | 0.33, 0.49 | 0.76 | 0.69, 0.82 | 0.59 | 0.49, 0.72 | 1.13 | 0.82, 1.52 |
| Windsor-Essex County | 0.60 | 0.45, 0.78 | 1.05 | 0.99, 1.10 | - | - | 1.00 | 0.93, 1.07 | 0.86 | 0.73, 1.00 | 0.12 | 0.04, 0.26 |
| York Region | 0.15 | 0.08, 0.24 | 0.06 | 0.044, 0.069 | 0.05 | 0.03, 0.08 | 0.01 | 0.002, 0.01 | 0.07 | 0.04, 0.12 | 0.09 | 0.02, 0.22 |

Ѳ

**Additional file 4**. Opioid-related death rates across Ontario’s 34 Public Health Units, 2022


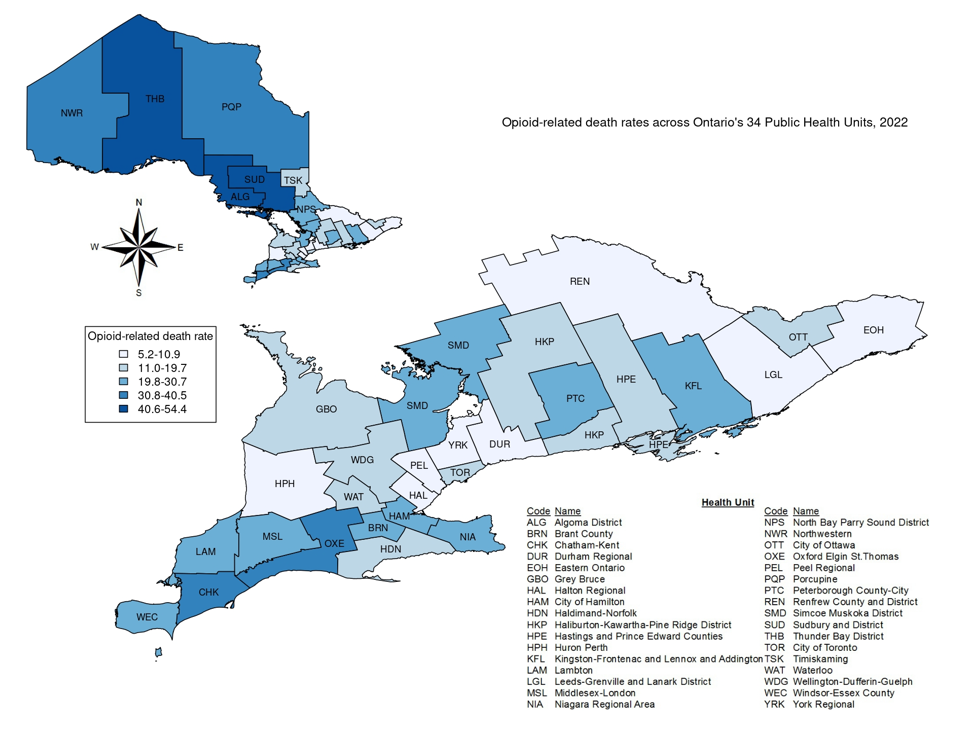

Supplement: Supplementary file 1 — Additional file. [file 12954_2025_1319_MOESM1_ESM.docx]
